# Modelling the structure of a ceRNA-theoretical, bipartite microRNA–mRNA interaction network regulating intestinal epithelial cellular pathways using R programming

**DOI:** 10.1186/s13104-018-3126-y

**Published:** 2018-01-12

**Authors:** J. M. Robinson, W. A. Henderson

**Affiliations:** Digestive Disorder Unit, Biobehavioral Branch, Division of Intramural Research, National Institute of Nursing Research, NIH, DHHS, Bethesda, MD USA

**Keywords:** MicroRNA, Competing endogenous RNA, Intestinal epithelial cells, Epithelial barrier function, Bipartite affiliation network, KEGG pathway database, Tight junction, Adherens junction, Regulation of actin cytoskeleton, Rac–Rock–Rho signaling

## Abstract

**Objective:**

We report a method using functional-molecular databases and network modelling to identify hypothetical mRNA–miRNA interaction networks regulating intestinal epithelial barrier function. The model forms a data-analysis component of our cell culture experiments, which produce RNA expression data from Nanostring Technologies nCounter^®^ system. The epithelial tight-junction (TJ) and actin cytoskeleton interact as molecular components of the intestinal epithelial barrier. Upstream regulation of TJ-cytoskeleton interaction is effected by the Rac/Rock/Rho signaling pathway and other associated pathways which may be activated or suppressed by extracellular signaling from growth factors, hormones, and immune receptors. Pathway activations affect epithelial homeostasis, contributing to degradation of the epithelial barrier associated with osmotic dysregulation, inflammation, and tumor development. The complexity underlying miRNA–mRNA interaction networks represents a roadblock for prediction and validation of competing-endogenous RNA network function.

**Results:**

We developed a network model to identify hypothetical co-regulatory motifs in a miRNA–mRNA interaction network related to epithelial function. A mRNA–miRNA interaction list was generated using KEGG and miRWalk2.0 databases. R-code was developed to quantify and visualize inherent network structures. We identified a sub-network with a high number of shared, targeting miRNAs, of genes associated with cellular proliferation and cancer, including c-MYC and Cyclin D.

**Electronic supplementary material:**

The online version of this article (10.1186/s13104-018-3126-y) contains supplementary material, which is available to authorized users.

## Introduction

**Fig. 1:**
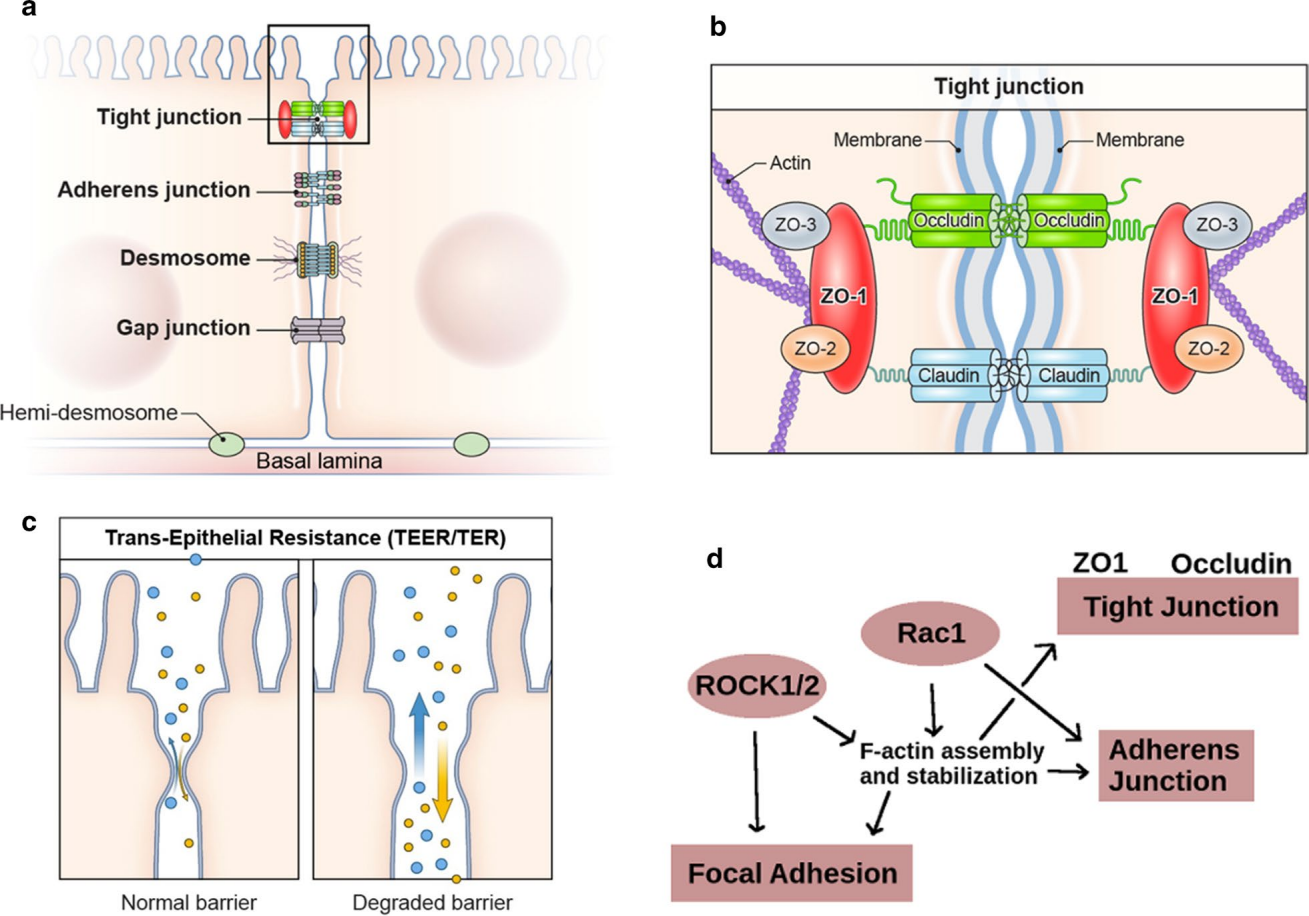
Rac–Rock–Rho pathway regulation of Cell–Cell junctions and actin cytoskeleton dynamics affect intestinal epithelial permeability. **a** Schematic of epithelial cell–cell junctions. **b** Detailed view of protein–protein interactions between the tight junction and actin cytoskeleton. **c** Degradation of intestinal epithelial barrier function allows solutes and macromolecules across the intestinal barrier. **d** Pathway structure of Rac–Rock–Rho activity affecting the actin cytoskeleton and cell–cell junctions

Increased intestinal permeability is associated with a variety of gastrointestinal disorders resulting from perturbation of intestinal epithelial homeostasis [1, 2], these include inflammation, chronic diarrhea [3] and Irritable Bowel Syndrome (IBS) [4, 5]. Epithelial barrier function is mediated by the tight-junction (TJ) complex, which maintains a barrier against paracellular translocation of macromolecules [6, 7] (Fig. 1a–c). TJs interacts with the epithelial actin-cytoskeleton; interactions are altered during actin cytoskeleton dynamic activity via influence from upstream activation from the Rac–Rock–Rho pathway [8–10]. Activation affects epithelial permeability and overlaps epithelial–mesenchymal transition (EMT) pathways [11, 12] (Fig. 1d). TJ-cytoskeleton pathways are also integrated with Wnt and Notch signaling and are associated with colorectal tumorigenesis [13].

**Fig. 2:**
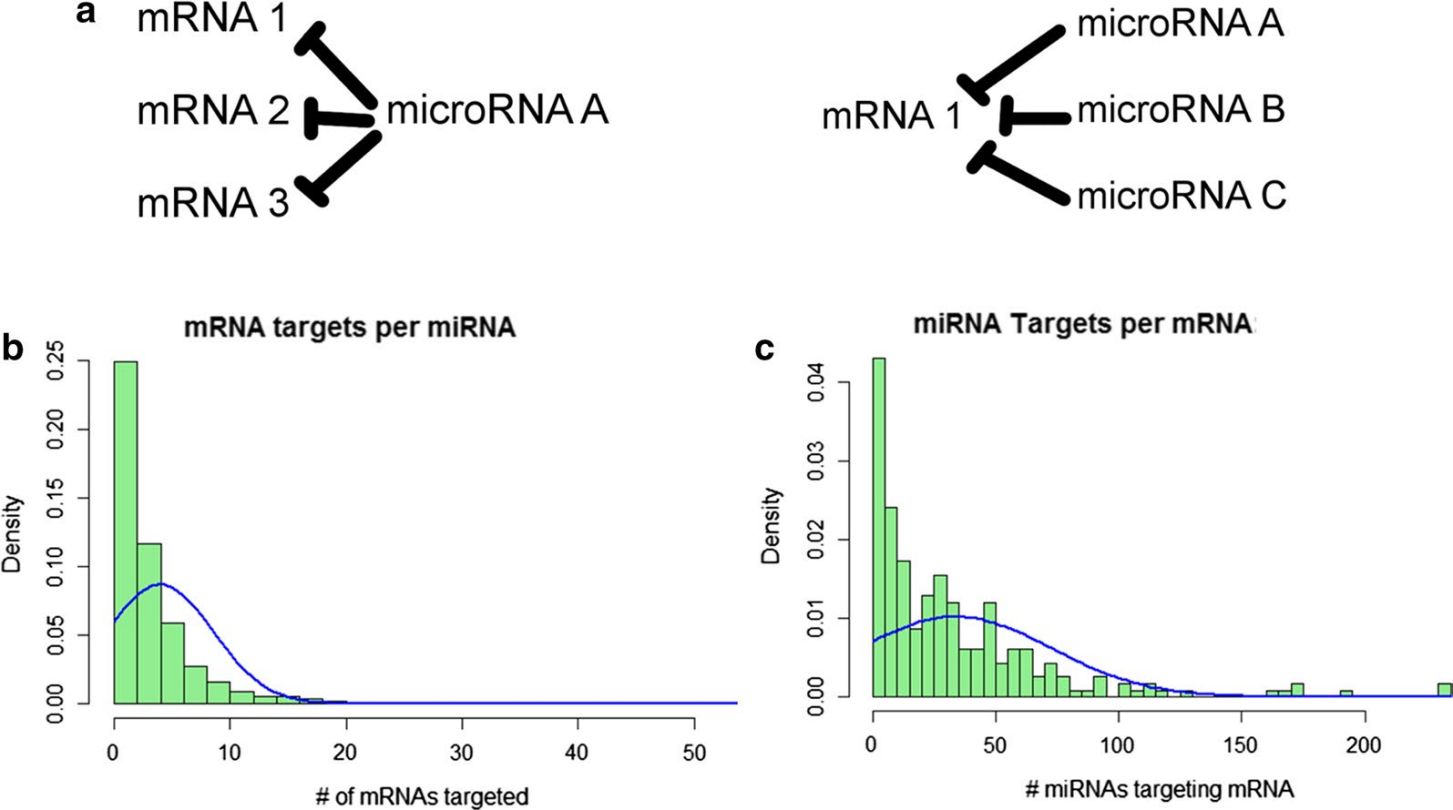
miRNA–mRNA targeting interactions form complex networks. **a** Individual microRNAs may target multiple mRNAs; an individual mRNA may be targeted by multiple miRNAs. **b**, **c** Frequency distributions for (**b**) mRNA target genes per miRNA (bin size = 2) and (**c**) Unique targeting miRNAs per mRNA (bin size = 5), according to the experimentally validated miRNA–mRNA interaction database miRWalk2.0

Another layer of regulatory complexity consists of post-transcriptional regulation involving microRNA (miRNA)–messenger RNA (mRNA) target interactions. In addition to specific miRNAs associated with intestinal homeostasis and permeability [14–17], miRNA–mRNA interactions result in competing-endogenous RNA (ceRNA) function. miRNAs may target multiple RNA transcripts, while an mRNA transcript may be targeted by multiple miRNAs (Fig. 2a), resulting in thousands of individual regulatory interactions with network-like effects on translation of co-targeted mRNAs. The ceRNA hypothesis was developed to describe such effects, however the full functionality and nuance of ceRNA is not fully understood [16–19]. Few bioinformatic methods have been developed to specifically address the functional complexity of such networks, and none have specifically investigated ceRNA networks of intestinal epithelial homeostasis pathways, which is our primary focus.

Published reports often compare transcriptional data with results of target-prediction algorithms to validate a subset of interactions as biologically active vs. inactive, and are usually specific to cell lines or tumor types [31, 32]. Our goal is to describe a ceRNA model based on fundamentals of graph theory. We adapted the method to improve reproducibility and interpretability of a network input by incorporating the network into a graph, a mathematical data structure representing networks as nodes and edges (links between nodes) [33]. The mathematically-defined nature of the graph object provides portability and scalability for any dataset, input being a simple list of associations. Modelling a miRNA–mRNA interaction network as a graph object allows patterns and associations within the graph to be described and quantified in a standardized manner. Application of functional transformations such as the single-mode projection of a bipartite graph, provides reproducible solutions and improves visual interpretation [33]. The R programming language provides open-source packages with easy functionality for handling graph objects [26].

## Main text

### Methods

#### Selection of a subset of key pathway mRNAs using KEGG pathway database

We selected approximately 200 protein-coding genes of interest to use in downstream expression profiling on the Nanostring^®^ platform. Protein-coding genes with overlapping membership in canonical KEGG pathways of interest were selected [20]. These included hsa04810 (Regulation of actin cytoskeleton), hsa04530 (tight junction), and hsa05210 (colorectal cancer). The gene list was referenced against the Human Protein Atlas [21] to eliminate mRNAs poorly expressed in gastrointestinal tissues. The list was further narrowed to select for genes present in two or more pathways of interest. Additional pathways include Adherens Junction (hsa04520), Focal Adhesion (hsa04510), Wnt (hsa04310) and Notch (hsa04330). A final list of 196 gene transcripts was used in subsequent analyses (Additional file 1: Table S1).

#### Selection of a comprehensive subset of microRNAs based on Nanostring Technologies^®^ human miRNA expression panel

miRNAs were selected based on membership in the Nanostring Technologies^®^ human miRNA v3 miRNA expression assay, with 800 human microRNAs chosen for known expression, disease association, biological relevance, and phylogenetic conservation between mammalian taxa [22]. miRNAs without database hits for experimentally validated miRNA–mRNA interactions were excluded from further analysis (see “Identification of miRNA–mRNA interactions using MiRWalk 2.0 database” below). 657 miRNAs were chosen (Additional file 1: Table S1).

#### Identification of miRNA–mRNA interactions using MiRWalk 2.0 database

We used the MiRWalk 2.0 database, which aggregates data from miRTarBase, PhenomiR, miR2Disease and HMDD databases, to obtain a list of experimentally validated gene-miRNA interactions [23]. We removed interactions for miRNAs not found in our selected list of 657 human miRNAs, removed genes without validated miRNA-target interactions, and removed redundancies for multiple experimental validations for a single miRNA–mRNA pair. The final output contained 3414 individual miRNA–mRNA interactions, which was used as an *adjacency list* for subsequent network analysis. (Additional file 1: Table S1).

#### Developing R code to create a network/graph plot for analysis and visualization of miRNA–mRNA interaction. (See Additional file 2: Network_Code.R file; Additional file 5: R-input, adjacency list)

Distributions for mRNA–miRNA target density were obtained from the adjacency list to identify genes by # of targeting miRNAs and miRNAs by # of genes they target (Additional file 3: Figure S1). Resulting graphical readout is too large for print purposes, so frequency distributions were created (Fig. 2b, c) to summarize the distribution of the number of miRNA targets per gene, and gene targets per miRNA. Frequency distributions represent a dimensionally ‘flattened’ version of the network object, and provide a basis for future comparison of different input lists.

**Fig. 3:**
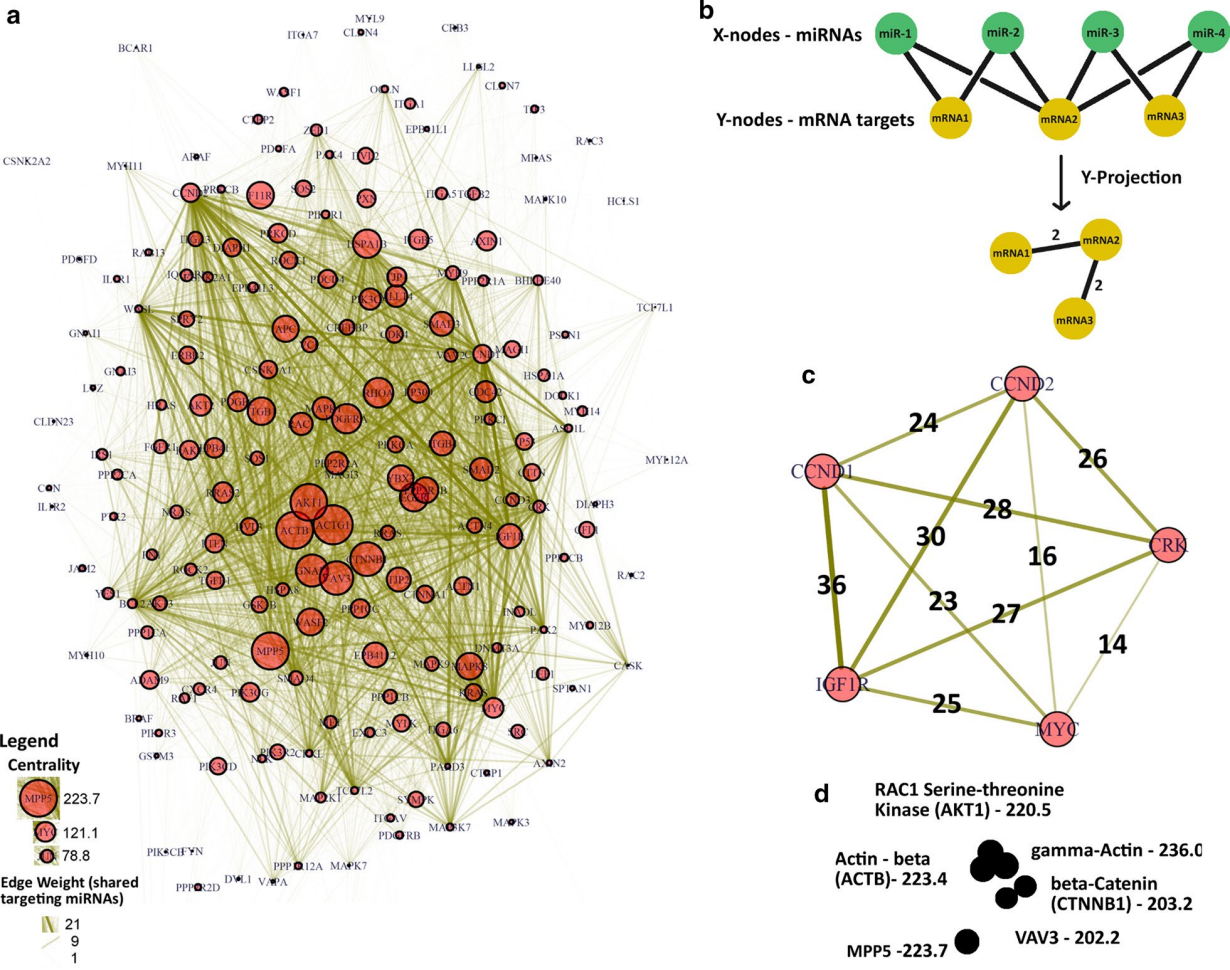
Network plot for visualization of high edge-weight sub-network interactions and centrality. **a** Network graph plot produced using miRNA–mRNA target list as an adjacency list, using the Additional file 2: R code provided in the supplement. Node size represents network Betweenness Centrality, and edge width and transparency represents edge weight as the number of shared, targeting miRNAs. **b** Bipartite affiliation networks such as miRNA–mRNA interaction networks can be projected as a single-mode with edge weights representing shared affiliations, as the network in **a**. **c** The 5 genes with highest number of shared, targeting miRNAs were subset and re-run in the R script with modified vertex and edge scaling (see additional comments in Additional file 2: Network_Code.R). The sub-network depicts a hypothetical ceRNA-functionality between these five genes, with edge-associated numbers equal to shared, targeting miRNAs between two genes, or nodes (See Additional file 6: Subset adjacency list). **d**. Genes with highest network centrality values (from the main network depicted in Fig. 3a graph where node size is scaled to network centrality), represent the most ‘central’ members of the overall miRNA–mRNA interaction network. Network centrality is a network-specific value: re-running this five-gene subnetwork in the R-script does not provide additional informative centrality information

We developed R code to use the miRNA–mRNA target interaction list (adjacency list) as a bipartite affiliation network, which is appropriate because of the network structure where mRNAs interact with miRNAs, miRNAs interact with mRNAs, but individual mRNAs and miRNAs do not interact with each other. R code was originally taken from open-source code for social network analysis and modified. We treated coding genes as ‘individuals’, and targeting miRNAs as ‘groups’ [24, 25]. R packages ‘Matrix’ and ‘igraph’ were used to convert the adjacency list into an adjacency matrix, and create a single-mode projection where the ‘mRNAs’ became nodes and edges represent shared, targeting ‘miRNAs’ [26] (Fig. 3b, Additional file 2: R code). Edge weights were defined values representing the number of shared miRNA–mRNA target interactions. The R matrix package performs the cross-products calculations where the number of shared, targeting miRNAs are converted into an edge-weight value between target mRNA nodes [27]. For example, in Fig. 3b a single ‘X-node’ (i.e., miR-1) interacting with two ‘Y-nodes’ (i.e., mRNA1 and mRNA2) in the bi-modal projection becomes a single edge between miRNA1 and miRNA2 in the single-mode projection, and adds value ‘1’ to the edge weight between miRNA1 and miRNA2. When two ‘Y-nodes’ share multiple interacting ‘X-nodes’, the single-mode edge weight becomes the number of shared, interacting ‘X-nodes’, which are removed from the single-mode projection. Edge-weight value becomes integrated into the mathematical graph object [26], igraph package and can be assigned to the plotted graph as edge-width and/or transparency values.

For the graph plot, edge width and transparency was assigned from edge-weight values. Vertex size was assigned from values for betweenness-centrality, a measure of shortest-path distance for nodes in a network [28]. Edge width and transparency, and vertex size, were modified to reduce the ‘hairball’: edge weight was transformed by a factor of .03 to obtain edge width, though the specific factor will vary between input networks to obtain the best resolution for visual interpretation.

### Results

The network contains 196 nodes representing genes from our pathways of interest (Fig. 3a). There are 7510 edges representing 20,807 shared miRNA-gene target interactions. The most well-connected sub-network consists of the genes Cyclin D1 (CCND1), Cyclin D2 (CCND2), Insulin-like growth factor 1 receptor (IGF1R), CRK proto-oncogene, adapter protein (CRK), and the transcription factor c-MYC (MYC). Edges between these nodes contain the highest numbers of shared, targeting miRNAs within the network, and forming a highly interconnected motif (Fig. 3c, Table 1). Interestingly, these are also the top-five most targeted genes in our network (Additional file 3: Figure S1).

**Table 1:** Pathway membership for selected network genes

Gene symbol	KEGG pathway membership					
Shared targeting miRNAs	Actin cytoskeleton	Tight junction	Colorectal cancer	Focal adhesion	Adherens junction	Wnt
CCND1	0	0	*1*	*1*	0	*1*
CCND2	0	0	0	*1*	0	*1*
CRK	*1*	0	0	*1*	0	0
IGF1R	0	0	0	*1*	*1*	0
MYC	0	0	*1*	0	0	*1*
Network centrality						
AKT1	0	*1*	*1*	*1*	0	0
ACTB	*1*	*1*	0	*1*	*1*	0
ACTG1	*1*	*1*	0	*1*	*1*	0
CTNNB1	0	*1*	*1*	*1*	*1*	*1*
MPP5	0	*1*	0	0	0	0
VAV3	*1*	0	0	*1*	0	0

Vertex (node) size was coded to correspond with the value for ‘betweenness centrality’ of that gene in the graph plot. Betweenness centrality is a measure of the shortest path distance within the overall network, essentially the nodes with the highest overall connectedness in the network [31]. These include gamma-actin (ACTG1), MAGUK p55 subfamily member 5 (MPP5), Actin-beta (ACTB), RAC-alpha serine/threonine-protein kinase (AKT1), beta-Catenin (CTNNB1), and vav guanine nucleotide exchange factor 3 (VAV3) (Fig. 3d, Table 1).

### Discussion

In the ceRNA hypothesis, co-regulatory effects occur when multiple genes are targeted by the same miRNA. Given steady state miRNA expression, increased expression of a one-target transcript creates additional miRNA target sites, acting as a ‘sponge’ for available miRNAs, resulting in decreased regulation across all targets of that individual miRNA [16, 17]. Manipulation of ceRNA networks is proposed route for novel therapeutics [29]. Many reports of ceRNA functionality are found in the literature, although the generality and context-dependence of ceRNA function is debated [18, 19, 30].

Our model makes specific use of the single-mode projection of a bipartite graph. A bipartite network has two sets of nodes, where nodes interact only with nodes of the opposite set. The basis of our model is that mRNAs and miRNAs form two sets of a bi-partite network. The complete network projection can be plotted so that nodes represent both sets of the bipartite network, and this is the most common ceRNA-network representation [31, 32]. The single-mode projection of a bipartite network facilitates easier visual interpretation, and gives quantitative readouts of graph properties (centrality, edge weight) [27, 33].

Using both the visualization, and distribution data derived from R output, we observed potential sub-network graphs of interest. The highly-interconnected relationships of the CCND1-CCND2-IGF1R-CRK-MYC sub-network predicts that these could participate in ceRNA-functional co-regulation, which would integrate insulin hormone signaling (IGF1R) with key cellular proliferation components (MYC, Cyclins D1 and D2, CRK), well-known for their association with cellular proliferation and cancer (Fig. 3c). Under the ceRNA hypothesis, differential overexpression of any individual gene is predicted to result in decreased miRNA regulation of in-network genes. For example, increased expression of CyclinD2 hypothetically lowers miRNA regulation across the sub-network. Assuming this results in increased CyclinD1, D2 (promoting G1-S phase transition), c-Myc (transcription factor regulating proliferation-associated genes), and IGF1R (increased sensitivity and activation of insulin-like growth factor pathway signaling) protein, increased proliferation may result. The genes are known to behave similarly in ER-positive breast tumors [34]. Altered expression resulting from the ceRNA mechanism is subject to feedback from regulatory pathways which may mitigate (or enhance) ceRNA effects, for example MYC overexpression appears to inhibit CCND1 and increase apoptotic potential in pancreatic cancer cells [35].

These genes are the most highly-targeted genes in the network (Additional file 3: Figure S1). This may represent a generalizable feature of ceRNA-networks, and it will be interesting to test if the most highly targeted genes in any given network always have the highest number of shared, targeting miRNAs. The high network centrality of genes such as ACTG1, ACTB, AKT1, and CTNNB1 is also interesting in that they are not the most highly targeted genes. They include the two primary forms of actin, b-actin (ACTB) and g-actin (ACTG1) (Fig. 3d). AKT1 (beta-catenin) is a key member in Wnt signaling and adherens junction pathways, and AKT1 is an important kinase in focal adhesion, colorectal cancer, and many other pathways. It is unknown if such high network centrality has a biological or functional significance.

## Limitations

1. Experimental validation is necessary to determine if the network exhibits ceRNA-function as predicted. Experimentally-validated miRNA–mRNA target interactions were used as input, however our model did not incorporate stoichiometric functions where target transcripts. Many transcripts including lncRNAs have multiple target sites for an individual miRNA gene. Further development will seek to incorporate and experimentally validate effects of multiple target sites. A preliminary model for such effects is provided. (Additional file 4: A Brief Model for ceRNA Effects Resulting from Differential Target-Site Availability.)
2. Expression levels of miRNA and mRNA transcripts have a significant effect on ceRNA function on the size of the co-regulatory effect on other transcripts. For example, if co-targeted transcripts are expressed at low levels, even relatively high fold-changes will not provide a significant co-regulatory ceRNA effects, and the opposite for highly-expressed transcripts. Additional weight factors will be adapted to model effects of relative transcript abundance.
3. ceRNA function is dependent upon additional regulatory contexts such as differential splicing affecting miRNA target sites, RISC functional modifications, and transcriptional regulation, ceRNA function could be overpowered or mitigated by regulatory inputs unaccounted for in this model.
4. Focus on specific pathways of interest introduces bias into the gene-set selected for this analysis. Our focus on intestinal epithelial permeability genes produces a bias toward related pathways. Other input bias may include over-representation of experimental results in the database. Further comparative, quantitative testing of input miRNA-target interaction sets and use of reference sets will be an important factor for describing and controlling for input-bias effects in the future.

## Additional files

**Additional file 1: Table S1.** Citation data for miRWalk2.0-derived experimentally validated miRNA-mRNA interactions.

**Additional file 2.** R-code. An R-programming language script with functional code to perform all analyses described in this article.

**Additional file 3: Figure S1.** High-resolution histograms showing gene and miRNA names associated with their respective target numbers. The reader may use this to identify genes with highest and lowest numbers of targeting miRNAs, and miRNAs targeting the most and least number of genes.

**Additional file 4.** Additional Model—Multiple Target Sites. A Brief Model for ceRNA Effects Resulting from Differential Target-Site Availability.

**Additional file 5.** R-input, adjacency list. A comma-delimited adjacency list of gene-miRNA interactions, used as input for the R-code.

**Additional file 6.** R-input, Fig3c subset adjacency list. A subset of the MiRWalk_Trimmed.csv adjacency list, used to derive the graph plot displayed in Fig. 3c.
