# Supplementary figures and images for: Modelling the structure of a ceRNA-theoretical, bipartite microRNA–mRNA interaction network regulating intestinal epithelial cellular pathways using R programming
Source: BMC Res Notes. 2018 Jan 12;11:19. doi: 10.1186/s13104-018-3126-y (PMC5766989; doi:10.1186/s13104-018-3126-y)

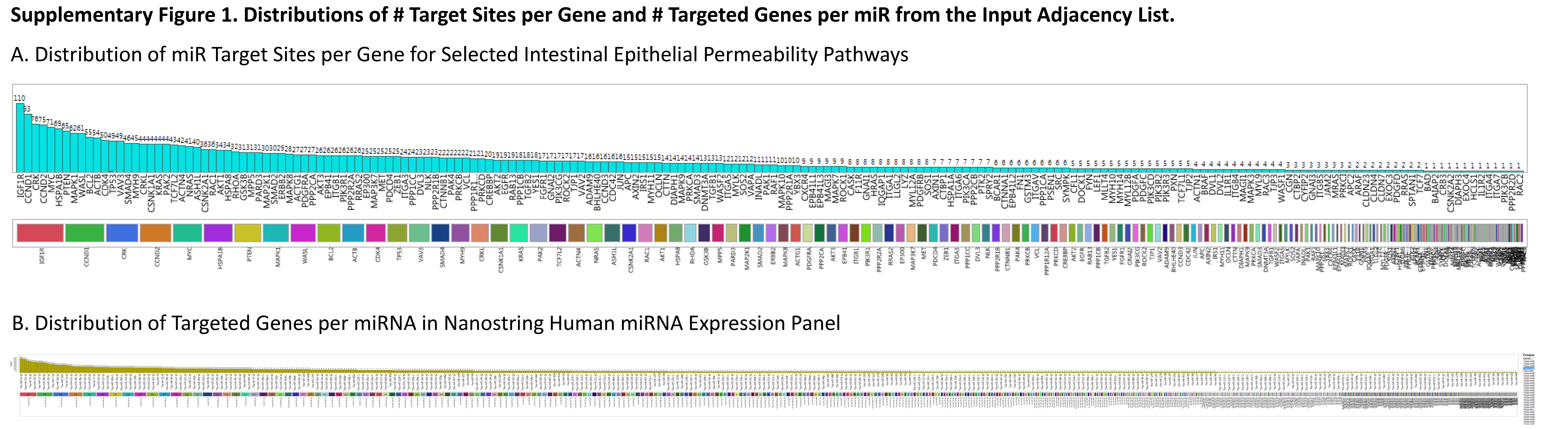

Supplement: Supplementary file 3 — Additional file 3: Figure S1. High-resolution histograms showing gene and miRNA names associated with their respective target numbers. The reader may use this to identify genes with highest and lowest numbers of targeting miRNAs, and miRNAs targeting the most and least number of genes. [file 13104_2018_3126_MOESM3_ESM.tif]
