# Supplementary material for: Modelling the structure of a ceRNA-theoretical, bipartite microRNA–mRNA interaction network regulating intestinal epithelial cellular pathways using R programming
Source: BMC Res Notes. 2018 Jan 12;11:19. doi: 10.1186/s13104-018-3126-y (PMC5766989; doi:10.1186/s13104-018-3126-y)

**Additional material – A Brief Model for ceRNA Effects Resulting from Differential Target-Site Availability.**

One limitation of our ceRNA model is the difficulty in accurately describing the role of multiple target sites for one miRNA on the same gene transcripts. Predictive algorithms are not particularly accurate in predicting biological responsiveness of specific target sites, either because the site is not bound by the RISC complex, or that the transcript is not differentially regulated even if the target site is bound. CLiP-seq and related methodologies have provided significant data on target-site binding, and account for a large proportion of the validated miRNA-gene target database MiRWalk2. However, the experimental data only provide target-interaction data for specific biological contexts (i.e., the cell-lines or tumor-types), therefore understanding the differential availability of target-sites and how variation would affect ceRNA function is an important goal.

We intend to incorporate the effects differential target-site availability into our model to gain a more realistic system for transcriptional data from our experimental studies, and to investigate whether a more accurate predictive result can be obtained from our experimental transcriptional data. Here we provide a basic outline for this modelling. This model is a prototype, and contains assumptions which may be false in an *in vivo* biological system, yet which could be modelled and validated given appropriate datasets.

1. **A Model for ceRNA Effects Under ‘ideal’ Regulation**
2. A simplified network with three genes, targeted by a single miRNA, each gene transcript has a single target site for the targeting miRNA. Assuming that all target sites have equal RISC binding and regulatory effect of 100% translational inhibition, and assuming a steady-state level of miRNA expression.
3. ceRNA effects will be the result of target availability, which will change based on differential expression of the three target transcripts.
4. In the first model, if three gene transcripts are expressed at 1000 copies, then there are 3000 total target sites. If there were an equal or greater than 3000 targeting miRNAs, target sites are saturated, therefore if one transcript (geneA) expression became 500 copies, no ceRNA effects would occur on geneB and geneC.
5. However, if gene A doubled expression to 2000 copies, there would be a total of 4000 target sites for 3000 miRNAs. This would cause differential ceRNA effects because transcript target sites would become unregulated.
6. The average ceRNA effect on an individual transcript can be described as the proportion of total target sites. 3000 miRNAs per 4000 target sites creates an inoccupancy of 1000 target sites, or an un-occupancy rate of .25.
7. Under these ideal conditions, 500 transcripts of geneA, and 250 transcripts each of geneB and geneC would become free for translation. In this scenario, increase in one gene (geneA), results in increases in geneB and geneC as well, though more highly expressed geneA has the expression advantage. Assumptions are that target-site occupancy results in ‘perfect’ translational regulation but not transcript degradation, and the steady-state counts of mRNA and miRNA transcripts were accurately known.
8. The unoccupancy rate of target sites could be used to provide a quantitative estimate of ceRNA effect.
9. **A Model for ceRNA Effects With Multiple Target Sites per Target Transcript**
10. Effects of multiple target transcripts are modelled using the concept of target-site occupancy rate. In this example, geneA, geneB, geneC are all expressed as 1000 transcripts, but geneA has two targets per transcript. With greater than 4000 targeting miRNAs, target-sites are saturated, so decreased expression of any target transcript does not affect regulation.
11. If there are exactly 4000 targeting miRNAs, and geneA expression increases by 500 transcripts to 1500 transcripts, then target-sites increase to 5000 total target sites. *Globally*, the target-site un-occupancy rate then becomes .2 (5000 target sites/4000 targeting miRNAs = .2; also 1.25 target transcripts per miRNA).
12. *Transcript occupancy* changes depending on target number per transcript. For example, geneA now has 3000 target sites per 1500 transcripts. miRNA-transcript un-occupancy for geneA becomes less because gene A has twice the target sites (2 target sites) per transcript than geneB and geneC, and is therefore twice as likely to be targeted (.2 target un-occupancy / 2 targets per transcript = .1 target unoccpancy per transcript). geneB and geneC would have 200 unoccupied targets sites and 200 unoccupied transcripts, resulting in (ideally) 200 expressed protein molecules per gene. geneA by contrast, with 600 unoccupied target sites (3000 x .2), split between 1500 *target transcripts*, creates .1 un-occupancy rate per target transcript, or 150 vs 300 unoccupied transcripts.
13. Target transcripts with a higher number of target sites for the same miRNA will remain more highly regulated under this model given a global increase in target sites assuming that one unoccupied target site has the same regulatory effect on individual transcript as two occupied target sites. It will become necessary to further model probabilistic molecular phenomena, for example, some transcripts of geneA may be more or less likely to have both or neither target site occupied. If all 600 unoccupied target sites were split between 300 geneA transcripts for example, then translational inhibition would be equivalent to geneB and geneC. Alternatively, if the 600 unoccupied target sites were distributed between 600 geneA transcripts, complete inhibition would be observed.


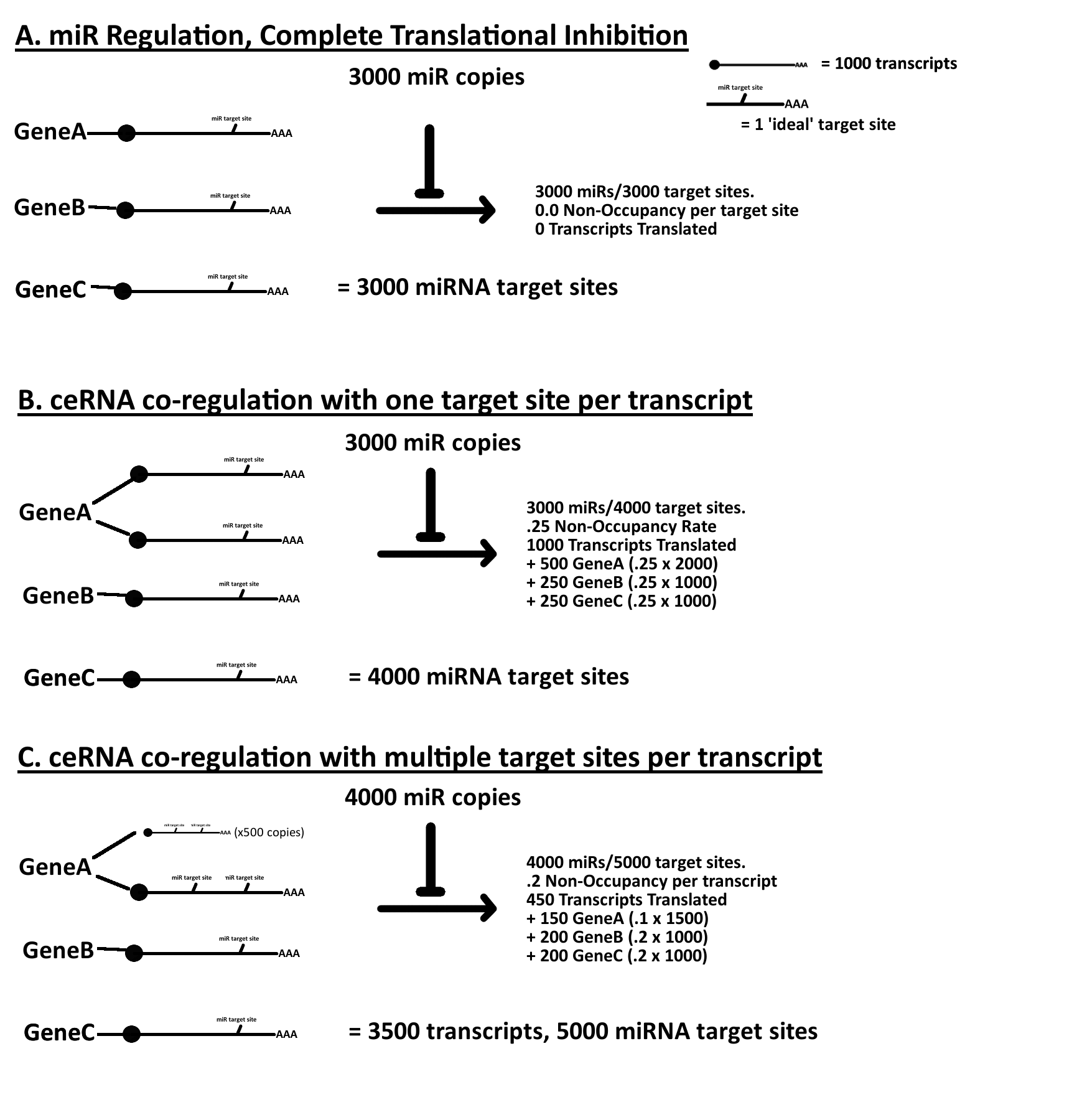

Supplement: Supplementary file 4 — Additional file 4. Additional Model—Multiple Target Sites. A Brief Model for ceRNA Effects Resulting from Differential Target-Site Availability. [file 13104_2018_3126_MOESM4_ESM.doc]
